# Influences of Ga Doping on Crystal Structure and Polarimetric Pattern of SHG in ZnO Nanofilms

**DOI:** 10.3390/nano9060905

**Published:** 2019-06-21

**Authors:** Hua Long, Ammar Ayesh Habeeb, Dickson Mwenda Kinyua, Kai Wang, Bing Wang, Peixiang Lu

**Affiliations:** 1 Wuhan National Laboratory for Optoelectronics (WNLO) and School of Physics, Huazhong University of Science and Technology, Wuhan 430074, China; I201622178@hust.edu.cn (D.M.K.); kale_wong@hust.edu.cn (K.W.); wangbing@hust.edu.cn (B.W.); 2 Physics Department of College Science, Diyala University, Baqubah 964, Iraq; ammarlaser72@yahoo.com; 3 Hubei Key Laboratory of Optical information and Pattern Recognition, Wuhan Institute of Technology Wuhan 430205, China

**Keywords:** ZnO nanofilms, SHG, Ga doping, polarization angle

## Abstract

The second-harmonic generation (SHG) in gallium doped ZnO (GZO) nanofilms was studied. The Ga doping in GZO nanofilms influenced the crystal structure of the films, which affected SHG characteristics of the nanofilms. In our experiments, a strong SHG response was obtained in GZO nanofilms, which was excited by 790 nm femtosecond laser. It was observed that the Ga doping concentrations affected, not only the intensity, but also the polarimetric pattern of SHG in GZO nanofilms. For 5.0% doped GZO films, the SHG intensity increased about 70%. The intensity ratio of SHG between the incident light polarization angle of 90° and 0°changed with the Ga doping concentrations. It showed the most significant increase for 7.3% doped GZO films, with an increased ratio of c/a crystal constants. This result was attributed to the differences of the ratios of *d*_33_/*d*_31_ (the second-order nonlinear susceptibility components) induced by the crystal distortion. The results are helpful to investigate nanofilms doping levels and crystal distortion by SHG microscopy, which is a non-destructive and sensitive method.

## 1. Introduction

Semi-conductor nanofilms are one of the most widely used nanomaterials due to their excellent properties. As one type of a range of widely used semiconductor materials, ZnO nanofilms are applied in the fields, including photo-voltaic devices, photo-catalysis, and bio-imaging for its characteristics, such as wide band gap and high transparency [1,2,3]. Doping can manipulate the optical and electrical components of the intrinsic ZnO materials. For instance, Ga-doped ZnO films have outstanding properties, such as the wider band gap and low reactivity. Because the radii of Ga and Zn atoms are similar, even at high doping concentrations, the Ga doping leads to a small lattice deformation in ZnO [4,5,6,7].

The non-linear optical properties of semi-conductor nanofilms have attracted a lot of attention due to their potential applications in nonlinear optical frequency converters, microscopic images, and all-optical communication. The developments in nonlinear optical techniques have opened a window into the morphological and structural characteristics for a variety of systems, even for biological systems. It can also be an alternative measurement scheme suitable for detecting dynamic processes. [8,9]. For instance, SHG is a very sensitive and non-destructive technique for several applications in various fields, such as crystal structural detection, cancer cell diagnostics, optical switches in nano-devices [10,11,12,13,14,15]. Previous studies have demonstrated highly efficient SHG in different semi-conductor nanomaterials, such as CdS, GaAs, ZnO [16,17,18,19,20,21,22]. Owing to its polarization sensitivity [23,24], the SHG method has been proven to be an optical method for the detection of crystal structures without damage or special environmental requirements. Furthermore, since the wavelength of pumping laser can be tuned conveniently, the SHG method is advantageous in identifying suitable wavelengths for different materials detection. Through tuning the pumping wavelength, this method can even be applied for depth-resolved detection within nanomaterials.

With the above advantages, the SHG method has been used to detect the crystal structure characteristics of the ZnO nanomaterials. Moreover, the efficient generation of SHG signal, from several ZnO nanostructures, has been reported. For instance, Neeman et al. reported crystallographic mapping of ZnO nanowires using the SHG method [25]. Han et al. also used the SHG microscopy method to detect the lattice distortion in a bent ZnO nanowire [26]. Much attention has been paid to the analysis of the crystal structure of nanomaterials using SHG signal. However, few studies have focused on the analysis of the crystal structure induced by doping, using polarimetric patterns of SHG in ZnO nanostructures. In this work, we investigated the Ga doping influences on the crystal structure and polarimetric pattern of SHG in ZnO nano-films. The deviation of crystal site symmetry in Ga-doped ZnO nano-films was also determined from X-ray diffraction (XRD) results, and it was found to be strongly correlated with the SHG polarity. We found that the SHG intensity increased by about 70% for 5.0% doped GZO films. The intensity ratio of SHG between incident light polarization angle of 90° and 0° changed with the Ga doping concentrations. This result was attributed to the differences of the ratios of d_33_/d_31_ induced by the crystal distortion. The results are helpful to determine nanofilms doping levels and crystal distortion using SHG method, which is a sensitive and non-destructive method.

## 2. Materials and Methods

The GZO films were deposited on silica substrates using pulsed laser deposition (PLD). The GZO targets with different Ga concentrations (0, 2.93%, 5.0%, 7.3%, 9.9%, and 20.9%, respectively) were fabricated [27,28]. To obtain the GZO targets, the powders of Ga_2_O_3_ (99.99%) and ZnO (99.99%) were mixed and sintered at 1350 °C for 48h. To clean the silica substrates, a mixed solution of H_2_SO_4_ and H_2_O_2_ was firstly used. The substrates were soaked in the mixed solution for 1 h at 80 °C. Then the substrates were treated with ultrasonic cleaning in another solution of H_2_O_2_, NH_4_OH, and H_2_O for 1 h. Excimer laser (Lambda Physik, KrF, 248 nm, 5 Hz, Coherent, Santa Clara, CA, USA) was used in the PLD. Before film deposition, the vacuum chamber was evacuated. The background gas pressure was 4.5 × 10^−3^ Pa. The oxygen gas was then introduced in the vacuum chamber. During the deposition the oxygen pressure was kept at 0.2 Pa. The substrate temperature was kept at 80 °C in the deposition process. The deposition time was 40 min. After that, the films were annealed for 1 h at 500 °C in air.

The surface morphology of the GZO films were measured through field emission scanning electron microscopy (FESEM, Sirion 200, FEI Co., Hillsboro, OR, USA). The crystal structures of the GZO films were determined by X-ray diffraction (XRD, PANalytical B.V., Almelo, Horland, the precision of 2θ is 0.00001°). Optical transmittance spectra of the samples were measured by UV-visible spectrophotometer (HITACHI U3310, Tokyo; Japan).

**Figure 1:**
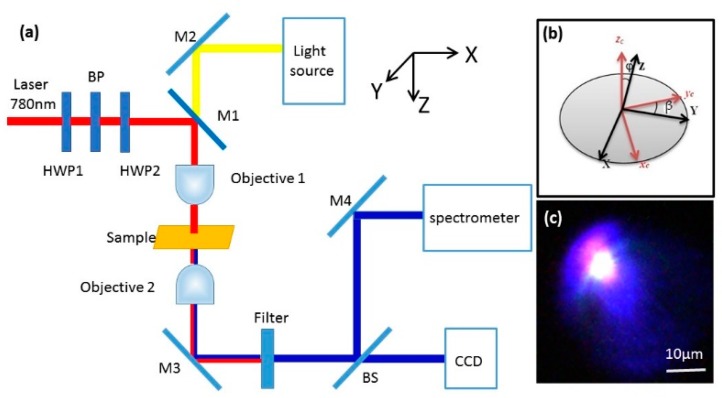
(**a**) Schematics of the experimental setup for measuring second-harmonic generation (SHG) signals; (**b**) geometry of the lab frame (*X*, *Y*, *Z*) and the crystal frame (*x_c_*, *y_c_*, *z_c_*). While, ϕ is the angle between the *z_c_* axis and the *Z* axis, *β* is the angle between the *Y* axis and the *y_c_* axis on the *xy* plane; (**c**) optical microscope image of the SHG of the ZnO film.

The SHG characteristics of the GZO films were detected by a home-built optical microscopy setup, as illustrated in Figure 1a. To pump the GZO films, the femtosecond laser beam from a mode-locked Ti-sapphire laser system (Tsunami, Spectra-Physics, 50 fs, 76 MHz, @790 nm, Newport Co., Irvine, CA, USA) was focused on the GZO nanofilms by a 40× objective (NA = 0.55) [29,30]. The transmitted SHG signals were collected by another 40× objective. Then, the transmitted signals were detected by a CCD or through a fiber coupling by a spectrometer (Acton 2500i, Princeton Instruments, Trenton, NJ, USA). In front of the CCD and the spectrometer, a 750-nm short-pass filter was used to filter out the incident laser. The laser power was measured by a laser power meter with a precision of 0.1 mW. In order to adjust the intensity of the pumping laser, we used a half-wave plate (HWP1) and a polarizing beam splitter (BP). Moreover, another half-wave plate (HWP2) was used to change the polarization direction of the pump laser. Figure 1b illustrates the crystallographic frame and the geometry of the laboratory frame. The laser propagated along the *z*-axis in the laboratory frame, whereas the films lied in the *xy* plane. Angle *β* determined the polarization of the incoming laser. Angle *φ* was 0°. Figure 1c shows the optical microscope image of the SHG signal of ZnO nanofilm detected by the CCD.

## 3. Results and Discussion

**Figure 2:**
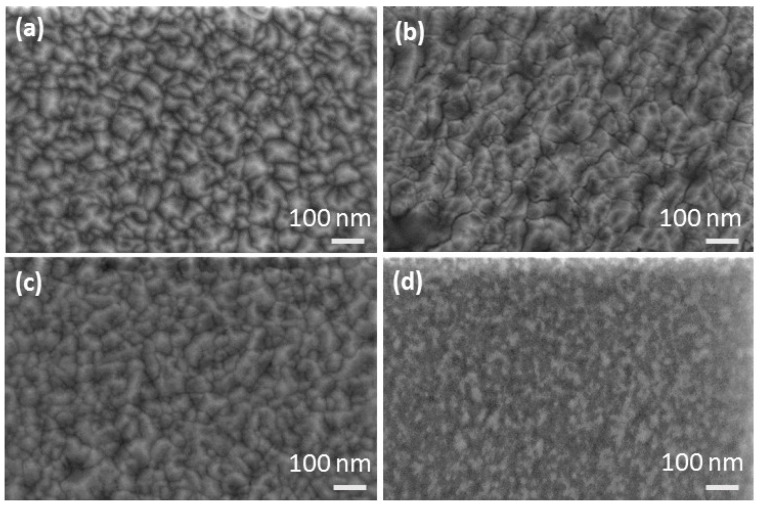
The SEM surface images of the gallium doped ZnO (GZO) films with different doping concentrations. (**a**) 0; (**b**) 5.0%; (**c**) 9.9%; (**d**) 21.9%.

The scanning electron microscopy (SEM) images of the GZO films surface (with doping concentrations of 0, 5.0%, 9.9% and 21.9%, respectively) were shown in Figure 2. The images show homogeneous and nano-crystalline films without laser-induced large particles on the surfaces. It can be observed that the grain sizes of the films were about several tens nanometers. And for the 21.9% doped ZnO film, the grain sizes show an obvious decrease.

**Figure 3:**
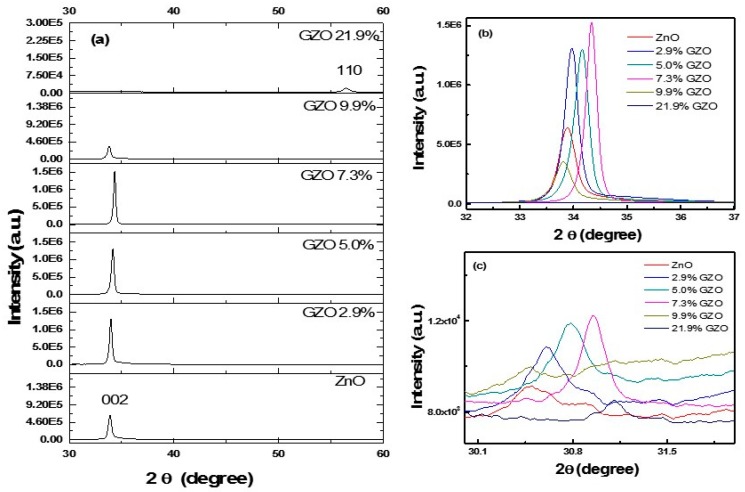
(**a**) XRD patterns of the GZO films; (**b**) the enlarged view of the diffraction peak of (002), showing the shifting of the diffraction peak position; (**c**) the enlarged view of the diffraction peak of (100), showing the shifting of the diffraction peak position.

To analyze the crystal distortion in ZnO nanofilms induced by doping, we compared the XRD patterns of the GZO films. As can be seen in Figure 3a, GZO films with hexagonal wurtzite structure were obtained. The XRD patterns show the characteristic diffraction peaks of the pure hexagonal wurtzite phase. For the undoped ZnO nanofilm, the diffraction peak of (002) at around 2θ = 34.0° was strong. And the diffraction peak at around 2θ = 30.5° corresponding to the wurtzite (100) peak was very weak. The strong (002) XRD peak indicates a high-quality crystal structure with c-axis preferred oriented. The preferential growth along c axis is due to the lower energy of growing along the [1] direction. Generally, the (0001) plane has the highest energy among all the facets. For instance, Zhan, et al., reported the (10$\bar{1}$0) plane has a surface energy of about 1.8 J m ^−2^. And the Zn-terminated (0001) surface has higher surface energy in the range from 2.5 to 5.8 J m^−2^. So according to Gibbs-Wulff theory, the growth along [1] direction leads to a preference of exposing low energy (10$\bar{1}$0) planes [31,32,33]. Then, due to the lower energy of growth along the [1] direction, the film showed preferential growth along *c* axis. GZO films, with lower doping concentrations (2.9%, 5.0%, 7.3% and 9.9%), showed similar XRD patterns as ZnO. While, for 21.9% Ga doped GZO films, only a weak diffraction peak at 2θ = 56.5° corresponding to (110) was observed. This was attributed to a significant decrease of crystal quality and a variation in the crystal structure.

The diffraction peaks around 2θ = 34° and 2θ = 30.5° of the GZO films were shown in details in Figure 2b,c respectively. We can see that the peaks gradually shifts to a level higher than 20 as Ga concentrations increased up to 7.3%. This shift indicates the reduction of the lattice spacing because the atomic radius of Ga (0.062 nm) is less than Zn (0.074 nm). When the Ga doping concentration was further increased to 9.9%, the two diffraction peaks shifted conversely towards lower 2θ. It might be induced by degradation of the crystal quality. Heavy doping Ga led to significant distortions and dislocations. For the GZO film with 21.9% Ga doped, no diffraction peak of (002) could be observed. A weak diffraction peak corresponding to (110) was observed. The heavily doped film were not grown along *c*-axis. The film grew with a preferred orientation along the (110) plane. These results were in agreement with the previous works [34,35,36].

From the XRD results, we are able to evaluate the grain size (D) using the following equation (Scherrer’s formula) [37]:

D = kλ/(β_0_cos(θ)) (1)

where β_0_ is the full width at half maximum (FWHM), k is a constant. The average grain sizes were calculated using this formula for GZO films. The calculated values are listed in Table 1. It can be noted that as the doping concentration increased to 7.3%, a slight increase in the grain size occurred. With further increase of the doping concentration, the grain size decreased.

The lattice constants (c and a) were also calculated for (002) and (100) plane from the XRD patterns. Table 1 show the calculated lattice parameters for GZO films. The calculated values of lattice spacing shows the reduction of the lattice constants in GZO nanofilms. The calculated values of c and a constants are close to the values of ZnO films previously reported [38,39,40]. The decrease of c and a constants with doping has been reported for GZO films fabricated by radio frequency magnetron sputtering method [41]. The observed reduction verifies the incorporation of Ga ions in ZnO lattice. The lattice reduction with the Ga doping concentration shows a significant reduction up to the 7.3% doped GZO nanofilms and then increases with further Ga doping. The incorporation of the Ga ions caused the reduction of the lattice, due to a smaller atomic radius of Ga than Zn. According to Vegard’s law, the Ga element was successfully doped into the ZnO lattice because the lattice constants decreased linearly as the Ga concentration increased up to 7.3% [41]. The 7.3% Ga doping level may be close to the saturation of Ga doping in ZnO using PLD methods [42,43].

We then evaluated the crystal structure deformation caused by Ga doping by calculating the ratio of the lattice constant (c/a) for the GZO nanofilms. We then compared them with the standard c/a ratio of the undoped ZnO, with the hexagonal wurtzite structure. From Table 1, we can see that the GZO nanofilm with 7.3% Ga doping concentration have the largest c/a ratio, which means that the lattice distortion along *c* axis is the largest. Similar behavior has been observed for Eu doped ZnO nanowires [32,44]. Clearly the increase in the c/a ratio can be attributed to the introduction of Ga ions. The presence of O vacancies can also influence the system. Furthermore, since the Ga ions are smaller than the Zn ions, Ga ions and O vacancies may form defect complexes. As a result, we can expect the formation of several lattice distortions in the GZO nanofilms with further increasing doping level, and hence degradation of crystal structure quality.

**Figure 4:**
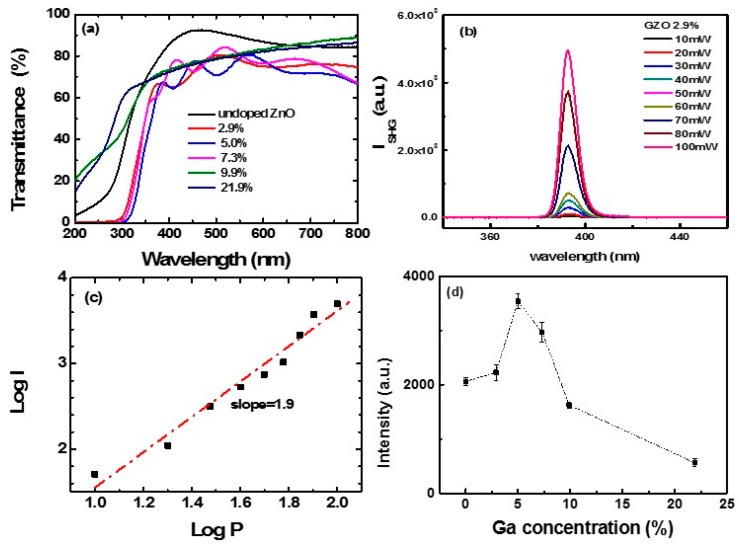
(**a**) Transmission spectra of the GZO films; (**b**) the spectra of SHG signal originated from 2.9% doped GZO film under different incident laser power; (**c**) the output SHG intensity as a function of the incident laser power. (**d**) SHG intensity changes as the Ga doping concentration increased. Dotted line is guide to the eye.

Figure 4a show the optical transmission spectra of the GZO nanofilms. As can be seen in the figure, the transmittance in the visible range is in the range of 70–90%. In the SHG experiments, strong SHG signal can be observed around 390 nm. Figure 4b shows the spectra of SHG for the 2.9% doped GZO film under different excitation laser power. Figure 4c shows the corresponding plots of SHG intensity versus the incident laser power in logarithmic scale. The slope of the fitting line is about 1.9. It confirms a quadratic response of the SHG intensity as a function of the exciting laser power, verifying a second-order nonlinear process [45,46]. And the measurements indicate a SHG susceptibility of ~15 pm/V at 810 nm [47,48]. The value is close to the reported values [49].

The influence of Ga doping on SHG intensity was investigated for GZO films. Figure 4d shows the SHG intensity (@ 390 nm) of the GZO films, with different doping levels, under the same excitation laser power. We can see that the intensity of transmitted SHG signal varied with gallium doping concentrations. It clearly indicates that the SHG intensity is sensitive to the doping concentration. The SHG intensity from the ZnO films improved by 5.0% and 7.3% in Ga doping. While, the SHG intensity showed a non-monotonic increase with the increasing Ga doping. The maximum values in SHG intensity were obtained in the 5.0% doped GZO films, which was about 1.7 times of the SHG intensity in the undoped ZnO films. We attributed the SHG enhancement to the influence of the centrosymmetry. Doping in the ZnO films significantly influenced the centrosymmetry of the ZnO, which could lead to the enhancement of the effective second-order susceptibility (*d_eff_*) in ZnO films and hence SHG effects. From the above crystal structure analysis, a notable modification of lattice constant in the GZO nanofilms was observed. For 7.3%, 9.9%, and 21.9%, the doped GZO nanofilms showed a decrease in SHG, compared with 5.0% doped GZO films. Similar results in the doping enhancement effects of SHG have been found in other materials, such as Eu doped ZnO nanowires, urea doped tristhioureazinc (II) sulfate, and oxalic acid doped ADP crystals [50]. Firstly, the SHG was enhanced with the increase in doping. However, with further increasing doping concentrations, the SHG enhancement effect decreased. We attributed this to the poor crystal quality induced by heavy doping and the influence of O vacancies. Furthermore, for heavy doped ZnO, the doped ions were segregated at the grain boundaries and not distributed in the crystalline matrix to enhance the non-linearity.

**Figure 5:**
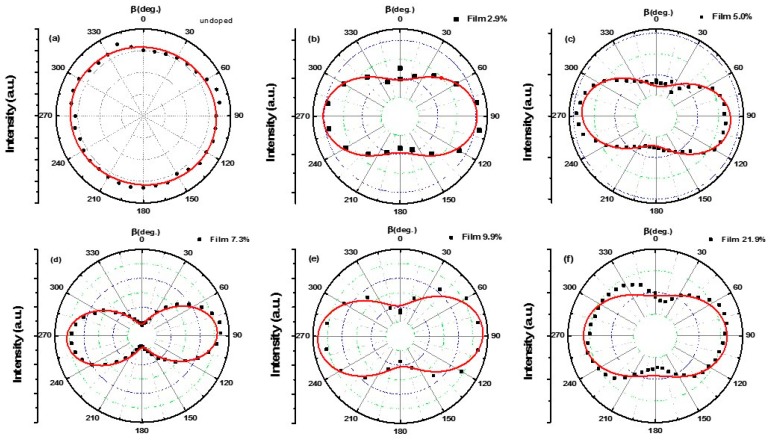
The normalized SHG intensity as the polarization angle, θ, changed for GZO films with different doping concentrations (**a**) 0; (**b**)2.9%; (**c**) 5.0%;(**d**) 7.3%; (**e**) 9.9%; (**f**) 21.9%. The experimental data are shown as dots and the solid curves show the theoretical fits.

The SHG intensity as a function of the excitation laser polarization and its dependence on the doping in GZO films were then studied. Figure 5a–f show the polar pattern evolution of the nanofilms with the increase in doping concentrations. From the figures, we can see that without doping, the SHG intensity I shows a circular shape. Once gallium was doped, the SHG pattern distorted significantly. The polar plots present a symmetrical shape of two-lobe. The apparent tilting of the lob in Figure 5c–e is attributed to the inaccuracy in the rotation of the polarizer in experiments. The dots indicate the experimental data and the red solid curves show the theoretical fitting. The fitting formula was as follows [51,52]:

*I* = *A*_0_(cos^2^*β* + *B*_0_sin^2^*β* + *C*_0_(sin*β*cos*β*))^2^ (2)

where *I* is intensity of SHG, *A*_0_, *B*_0_ and *C*_0_ are fitting parameters, *β* is polarization angle. The *c* axis of ZnO nanofilm is along the *z*-direction (shown in Figure 1b). Then for the *c*-axis oriented ZnO films, the second harmonic generalization electrical field, *P_x_*, *P_y_*, *P_z_*, can be described by the following matrix [24],

(3) $$\left[\begin{array}{c} P_{x}\\ P_{y}\\ P_{z} \end{array}\right]=\left[\begin{array}{cccccc} 0 & 0 & 0 & 0 & d_{15} & 0\\ 0 & 0 & 0 & d_{15} & 0 & 0\\ d_{31} & d_{31} & d_{33} & 0 & 0 & 0 \end{array}\right]\left[\begin{array}{c} \begin{array}{c} E_{x}^{2}\\ E_{y}^{2}\\ E_{z}^{2} \end{array}\\ 2E_{y}E_{z}\\ 2E_{x}E_{z}\\ 2E_{x}E_{y} \end{array}\right]$$

where *E_x_*, *E_y_* and *E*_z_ are the incident electric fields in the direction of *x*, *y,* and *z*, respectively. And *d_ij_* is the contracted notation of the second-order susceptibility tensor, which is a 3 × 6 matrix. The angle between the y and *y_c_* direction is defined as the polarization angle, θ, see Figure 1b. The obtained patterns are relative to the characteristic of the wurtzite crystal structure.

In our experiments, the objective with NA 0.55 were used to collect the SHG signal. The corresponding collective angle was about 30 degree. So the SHG signal detected was caused by *P_x_*, *P_y_* and *P_z_*, the whole intensity can be indicated as following,

*I*∝*P_x_*^2^ + *P_y_*^2^ + *P_z_*^2^ (4)

Due to the centrosymmetry, the polarimetric pattern of undoped ZnO shows a circular shape. When the doping concentration in ZnO nanofilms increased, the crystal structure changed, as confirmed by the XRD results. As a consequence of this structural change, the *d_ij_* components change accordingly by doping. According to the reference [53], the s-polarized and p-polarized SHG signal can be fitted as following,

*E_s_*^2*ω*^∝*Ad*_15_cos*β*sin*β* (5)

*E_p_*^2*ω*^∝(*Bd*_31_ + *Cd*_33_ + *Dd*_15_)sin^2^*β* + *Ed*_31_cos^2^*β* (6)

where *E_s_*^2*ω*^ and *E_p_*^2*ω*^ are the *s*-polarized and *p*-polarized SHG electrical field, separately; A–E are fitting parameters, which are related to the Fresnel transmission coefficients for fundamental beams and the corresponding refraction angles in the film for the fundamental and second harmonic wavelengths. Because *d*_33_ is much larger than *d*_31_ and *d*_15_ in ZnO, we conclude that the intensity ratio (between *β* is 90° and 0°) is most related to *d*_33_*/d*_31_ [49,54,55].

**Figure 6:**
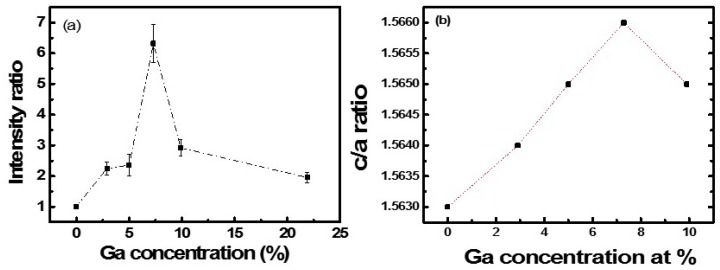
(**a**) Plot of the SHG intensity ratio between β = 90° and 0° (black dots) for GZO films as a function of Ga doping; (**b**) Plot of c/a ratio for GZO films as a function of Ga doping. Dotted lines are guide for the eye.

In order to analyze the relationship between the dependence on the incident laser polarization and the crystal distortion induced by Ga doping, the intensity ratio of SHG between *β* = 90° and 0° in GZO nanofilms was plotted, see Figure 6a. Obviously, the intensity ratio showed a dramatic increase as the doping in ZnO nanofilm increased to 7.3%. To further increase the doping concentration, the ratio decreased. At the same time, the variation of c/a ratio in the doped GZO nanofilms, estimated by XRD, was shown in Figure 6b, which shows a similar trend with the variation of the SHG intensity ratio. We also noticed that the shift of the diffraction peak (Figure 3b) in the nanofilms was in the range from 0.1° to 0.4°. The corresponding variation of the lattice spacing was estimated to be in the range of 0.01 nm to 0.06 nm. The results indicate that crystal distortion can significantly affect the second order susceptibility tensor. The intensity ratio of SHG between *β* = 90° and 0° in GZO nanofilms is related to the absolute value of *d*_33_/*d*_31_. It increased as the doping concentration increased up to 7.3% at doping, with the corresponding increase in the c/a ratio.

Therefore, the polarization-dependent SHG method can be used as a sensitive and non-destructive detection method for the crystal distortion induced by doping. Compared with traditional XRD methods, it is compatible with various experiment conditions, such as in atmosphere or in liquids. The results are also helpful for sensitively detection of the doping level using the optical method.

## 4. Conclusions

The crystal structure distortion and SHG from GZO nanofilms, induced by Ga doping, were studied. The results show that the intensity ratio of SHG between *β* = 90° and 0° shows a similar trend with the change of c/a ratios induced by Ga doping. For 5.0% doped GZO films the SHG intensity increased about 70%. The results indicate that the polarization-dependent SHG method has potential application as a non-destructive and sensitive detection of the crystal structure distortion induced by doping. Compared with XRD, it can be compatible with different experimental conditions. These results are also helpful for the sensitive detection of the doping level using the optical method.

## Figures and Tables

**Table 1:** Grain size and lattice parameter of Ga-doped ZnO thin films.

Doping Concentration (at. %)	FWHM	2θ (Degree)	D (Grain Size) (nm)	c (Å)	a (Å)	c/a
0.0	0.326	33.91	25.47	5.285	3.381	1.563
2.9	0.197	34.02	42.17	5.273	3.370	1.564
5.0	0.211	34.18	39.37	5.245	3.350	1.565
7.3	0.154	34.33	53.71	5.219	3.332	1.566
9.9	0.253	33.81	32.78	5.296	3.383	1.565
